# Overcoming barriers to polio eradication in challenging subnational geographies: lessons from South Khyber Pakhtunkhwa, Pakistan, 2022–2024

**DOI:** 10.3389/fpubh.2026.1768343

**Published:** 2026-04-21

**Authors:** Hafizullah Khan, Shabnum Khawas, Mohammad Wajid Ali, Ahmad Ameen, Abdul Basit, Muhammad Hakim, Adekunle Akerele, Richard Franka, Abdinoor Mohamed

**Affiliations:** 1 National Stop Transmission of Polio (NSTOP) Program, Islamabad, Pakistan; 2 Data and Analytics Team, Integral Global, Islamabad, Pakistan; 3 Khyber Pakhtunkhwa Provincial Government, Health Department, Peshawar, Pakistan; 4 Division of Global Immunization, Centers for Disease Control and Prevention, Atlanta, GA, United States

**Keywords:** community engagement, global polio eradication, insecurity and conflict, Pakistan, routine immunization, South Khyber Pakhtunkhwa

## Abstract

The global polio program has relied on supplementary immunization activities (SIA) in countries where routine immunization (RI) coverage is suboptimal. During these SIAs, oral poliovirus vaccine (OPV) is administered to all children under the age of 5 years. However, implementing SIAs in areas with insecurity and conflict, such as the South Khyber Pakhtunkhwa (South KP) region of Pakistan is very challenging because of attacks against vaccination teams and community boycotts among other reasons. These challenges sometimes lead to cancelation, delays, and poor quality of immunization campaigns. We conducted a retrospective secondary data analysis using Pakistan polio program data sourced through National Polio Emergency Operations Center’s dashboard, the Expanded Program on Immunization’s Management Information System, and the World Health Organization’s Immunization Data and Information Management System (IDIMS) to document the different vaccination strategies implemented in the South KP region of Pakistan from January 2022 to December 2024. During this period several SIAs were canceled in the region. In addition, SIAs in South KP were not synchronized with the rest of the province and country, and the duration of these campaigns were sometimes shortened. To address these challenges, several context specific novel interventions were initiated including mobile vaccination teams, ring-fencing vaccination around insecure areas, establishment of permanent transit vaccination points, and closer integration with routine immunization services through targeted use of fractional dose inactivated poliovirus vaccine, identification of zero-dose children during polio SIAs, extended outreaches, and provision of integrated health services through community health camps. Findings from South KP show that strategic alignment between polio and RI programs, flexible operational modalities, and locally responsive engagement can sustain vaccination services despite ongoing insecurity.

## Introduction

The Global Polio Eradication Initiative (GPEI) launched in 1988, successfully reduced poliomyelitis cases by more than 99% from 350,000 in 1988 to 99 cases globally in 2024, and two of the three wild poliovirus (WPV) serotypes (2 and 3) have been eradicated (1). Despite efforts, including huge resource mobilization, advocacy with governments, establishment of sensitive surveillance systems and improvements in population immunity through routine immunization (RI) and supplementary immunization activities (SIAs), the GPEI’s goal to interrupt transmission of all wild polioviruses by December 2023 (2) was not met. Endemic transmission of WPV serotype 1 (WPV1) continues only in Pakistan and Afghanistan, with outbreaks linked to importation of virus from Pakistan occurring in Southern Africa: one WPV1 case from Malawi in 2021 and eight WPV1 cases from Mozambique in 2022 (3, 4).

For polio, Pakistan and Afghanistan form a single epidemiological block with deep social, cultural, and economic ties and large-scale cross-border population movements. Both countries face security challenges, with over 2,600 kilometers of a porous border, often exploited by militant groups. To achieve permanent interruption of WPV1 transmission, it is crucial to reach high population immunity across both countries simultaneously through vaccination of every child with both oral poliovirus vaccine (OPV) and inactivated poliovirus vaccine (IPV).

Over the years, the global polio program has relied on SIAs in countries where RI coverage is suboptimal. During these vaccination campaigns, OPV is administered to all children under the age of 5 years. OPV administration not only protects children against paralysis, but also induces mucosal immunity that reduces person-to-person transmission of poliovirus and helps to interrupt circulation (5). SIAs aim to reach children who have not been vaccinated through RI and boost the immunity of those who have been vaccinated. Over the past two decades, polio SIAs have delivered approximately 10 billion doses of OPV to children worldwide, contributing to increased population immunity against polio to levels high enough to successfully interrupt WPV transmission in five of the World Health Organization (WHO) six regions (6–8). However, implementing SIAs in areas with insecurity or conflict, such as Khyber Pakhtunkhwa (KP) province of Pakistan, South-Central Somalia, Northern Nigeria and the Eastern and Southern provinces of Afghanistan, is very challenging and population immunity has not reached adequate levels to interrupt transmission (9). These conflict-affected areas face disruptions to vaccine delivery, inability to conduct optimal surveillance for poliovirus, and threats to the safety and security of health workers, among other challenges. The GPEI has employed a variety of innovative strategies adapted to specific country social context to improve vaccine uptake in conflict-affected areas (10). Examples of these strategies include community engagement, tailored communication strategies, and involving leaders and local influencers to enhance vaccination acceptance; setting up rapid response capabilities and robust surveillance systems at the local level, and using innovative approaches to deliver vaccines at transit points and at health camps together with other preventative and curative health services (11).

Eliminating polio in Pakistan and Afghanistan is the final hurdle to achieve a polio-free world, a major milestone in global public health. The South KP region is one of the major reservoirs of WPV1 in Pakistan. Following a significant decline of WPV1 paralytic cases in 2021, South KP reported 100% (20/20) of the Pakistan total cases in 2022, 50% (3/6) in 2023, and 24% (18/74) in 2024 (12). Absence of health facilities, very low RI coverage and suboptimal implementation of polio SIAs are the major reasons for persistent poliovirus circulation in South KP. Implementing polio SIAs has become increasingly challenging because of direct attacks against vaccination teams, vaccine mistrust and community demand for other governmental services (13).

This study describes various vaccination strategies implemented in South KP to address specific community challenges and close immunity gaps to polio and other vaccine-preventable diseases. It also highlights successful practices and lessons that can be adapted to other settings where vaccination efforts are hindered by conflict and community mistrust.

## Context

### South KP region

The South KP region of Pakistan, includes Bannu and Dera Ismail Khan (D. I. Khan) divisions. Bannu division has three districts: Bannu, Lakki Marwat, and North Waziristan. D. I. Khan division has four districts: D. I. Khan, Tank, South Waziristan Upper, and South Waziristan Lower. The region is spread over 28,229 square kilometers bordering Afghanistan to the west, Pakistan’s Punjab province to the east and Baluchistan province to the south. This region includes the former Federally Administered Tribal Areas (FATA), a semi-autonomous tribal territory that existed from 1947 to 2018, when it was merged with KP province following an amendment to the Pakistan constitution. South KP is afflicted by high poverty rates, low literacy levels, and limited healthcare infrastructure, complicating the delivery of polio immunization services. Conservative culture and tribal norms in this region further influence healthcare-seeking behavior, especially for women and children.

According to the 2023 Census, the South KP region had a total population of 6,280,857 residing in 934,313 households. An estimated 1,089,191 children under 5 years of age were targeted for OPV supplementary immunization activities (SIAs), while 242,324 children under 2 years of age were targeted for routine immunization (RI).

The districts of South KP face interconnected security, geographic, and operational challenges that significantly affected the polio and RI operations. D.I. Khan and Tank districts have intermittent security incidents, scattered rural and riverine populations. Bannu district experiences spillover insecurity from North Waziristan district with influx of internally displaced people, and persistent community resistance leading to boycotts and refusals. Lakki Marwat district is characterized by targeted security threats, vast rural spread, and poor road infrastructure. South Waziristan and North Waziristan present the most complex environment, marked by a high-risk security context and tough mountainous terrain, repeated community boycotts and reliance on special operational modalities. These challenges contribute to persistently missed children and constrained campaign implementation.

### Data sources and analysis

We analyzed retrospective secondary Pakistan polio program data to document the implementation various vaccination strategies in South KP from January 2022 to December 2024, during which the region remained a WPV-1 endemic region within a polio-endemic country. Data sources included the Pakistan’s National Polio Emergency Operations Center (EOC) Dashboard, the Expanded Program on Immunization’s (EPI) Management Information System (MIS), WHO’s Immunization Data and Information Management System (IDIMS) and supplementary documentation from District Health Offices and District EOCs. Descriptive statistics were used to assess campaign quality (percentage of lots passing LQAS surveys) and program reach (number of missed children during campaigns, and number of zero-dose and unimmunized children reached through targeting hard-to-reach populations). Descriptive statistics and trend analysis were done using Microsoft Excel software (Microsoft Corporation, Redmond, WA, USA, version 2021) and geospatial analysis using QGIS software (QGIS Development Team, version 3.36.1).

This activity was considered an evaluation of polio program activities, deemed non-human research after review by US CDC and was conducted after receiving approval from Khyber Pakhtunkhwa Provincial Government through the Provincial EOC (see Table 1).

**Table 1:** Operational definitions for vaccination strategies and monitoring activities in South Khyber Pakhtunkhwa (KP), Pakistan, 2022–2024.

Operational definitions	
Supplementary immunization activities (SIAs)	Mass vaccination campaigns targeting children <5 years in areas with low RI coverage or outbreaks to supplement RI. For polio, this is conducted under the patronage of the Global Polio Eradication Initiative (GPEI).
Zero-dose children	Children under 2 years of age who have not received any type of polio vaccine either through RI or SIAs.
PEI-EPI synergy	Initiatives implemented through a collaboration of the Polio Eradication Initiative (PEI) and the Expanded Program on Immunization (EPI) to reduce under-vaccinated and zero-dose children.
Reaching the Unreached (RUR) activities	One of the PEI-EPI synergy initiatives that delivered multiple vaccine antigens in union councils (UCs) with significant immunity gaps and suboptimal RI and SIA coverage. These activities focused on reaching children <2 years who have missed one or several doses of RI vaccines.
Biker teams	Mobile vaccination teams that track and vaccinate high-risk mobile children using motorbikes. They deliver multiple antigens to zero-dose, due and defaulter children <2 years and OPV to all children < 5 years of age.
Ring fencing vaccination	Vaccination points established at key entry and exit points around security-inaccessible areas, aiming to identify and vaccinate children below <10 years old entering and exiting those areas.
Security-led SIA modality	SIA implementation strategy where the scope and timing of campaigns change depending on threat assessments by law enforcement agencies.
Directly observed vaccination (DOV)	A strategy to ensure genuine vaccination by having an independent observer verify the vaccination process, aimed at eliminating the practice of fake finger marking.
Lot quality assurance sampling (LQAS) methodology	A rapid survey method to provide information on whether a vaccination campaign has achieved a desired level of performance (quality) in a specified geographic area (lot). In Pakistan, each UC is considered a lot.
Transit points vaccination	Vaccination posts at key transit locations such as bus stands, markets, highways, and border crossings that provided OPV to children below 10 years belonging to mobile populations.
Health camps vaccination	Initiative implemented to bridge gaps resulting from suboptimal healthcare delivery and community resistance under the oversight of the KP provincial health department.
Extended outreach activities (EOAs)	Initiatives implemented by the EPI team and supported by the PEI between campaigns to increase RI coverage, targeting zero-dose and defaulter children identified in SIAs.
Big catch up (BCU)	Global initiative to restore RI coverage and close the immunity gaps caused by pandemic related disruptions to vaccination services.

### Immunization services in South KP

Children in Pakistan, including those in South KP, receive four doses of OPV through RI starting with a birth dose followed by doses at 6 weeks, 10 weeks and 14 weeks. Similarly, children receive two doses of IPV: one at 14 weeks and the other at 9 months. Immunization services in South KP are characterized by low RI coverage and suboptimal quality of polio SIAs. A Third-Party Verification of Immunization Coverage Survey (TPVICS) conducted in 2022 revealed that only 61% of children were fully immunized with all recommended vaccines, 29% were partially immunized, and 10% had received no vaccines. District-wise coverage rates for the third dose of pentavalent vaccine (Penta-3) were 77.3% in D. I. Khan, 68.1% in Bannu, 56.2% in Lakki Marwat, 35.1% in Tank, 25.6% in South Waziristan and 14% in North Waziristan (14).

Polio SIA implementations in South KP also faced significant challenges ranging from attacks against vaccination teams, community boycotts to extreme security incidents sometimes leading cancelation, delay and poor quality of campaigns. Between January 2022 and December 2024, at least 19 OPV SIAs had been planned for South KP, including 8 national immunization days (NIDs) and 11 sub-national immunization days (SNIDs). However, South Waziristan Upper district canceled five SIAs, D. I. Khan and Tank districts canceled three SIAs each, North Waziristan district canceled two SIAs, and South Waziristan Lower district canceled one SIA. In addition, in Bannu district, vaccination campaigns did not take place in three union councils (Jani Khel, Hindi Khel, and Sain Tanga) during three consecutive SIAs (November 2023, January 2024, and February 2024). In South Waziristan Upper district, a community boycott resulted in the cancelation of all campaigns for 16 months from July 2022 to November 2023. The number of children who missed vaccination in these canceled campaigns ranged from 35,000 (3%) to 714,000 (65%) of the total target population of children under 5 years old in the region.

**Figure 1:**
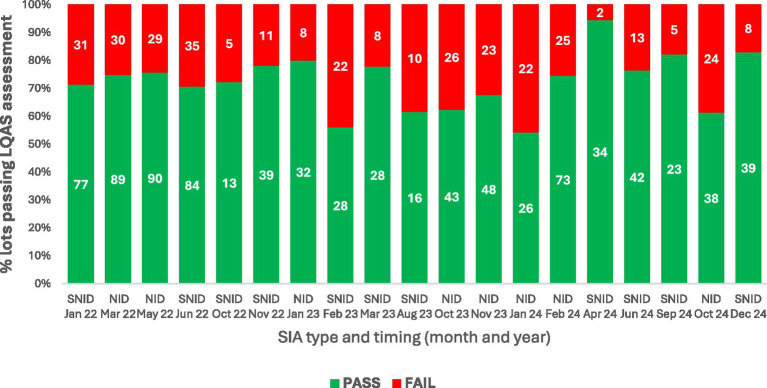
Proportion of lots passing LQAS assessment in Supplementary immunization activities (SIAs) conducted in South Khyber Pakhtunkhwa, Pakistan, 2022–2024. NID, National Immunization Days which are nationwide polio immunization campaigns; SNID, Sub-national Immunization Days which are polio immunization campaigns targeting high risk sub-national geographies.

For the campaigns that were not canceled, administrative data from the National Polio Program suggest that only a small proportion of target children missed vaccination. The average proportion of missed children was 1.5% ± 0.4 in the six polio campaigns conducted in 2022; 2.7% ± 1.3 in the six campaigns conducted in 2023; and 2.2% ± 0.7 in the seven campaigns conducted in 2024. However, data from post-campaign LQAS surveys pointed to a larger number of children missed in most campaigns. Of the LQAS surveys conducted from January 2022 to December 2024, only four out of 19 campaigns achieved the expected national standard of 80% of lots achieving a pass at ≥90% coverage; the other 15 campaigns had between 54% and 78% lots passing at ≥90% coverage (Figure 1). An additional limitation to the information on quality of vaccination activities during SIAs, was that many of the planned LQAS assessments (50% of the total UCs of South KP as per policy) could not be conducted for security reasons. In South Waziristan Upper district LQAS surveys were conducted only once in January 2024 after resuming SIAs activities in November 2023, and only 2 (5.9%) UCs could be surveyed.

### Changes in SIA implementation in response to challenges

**Figure 2:**
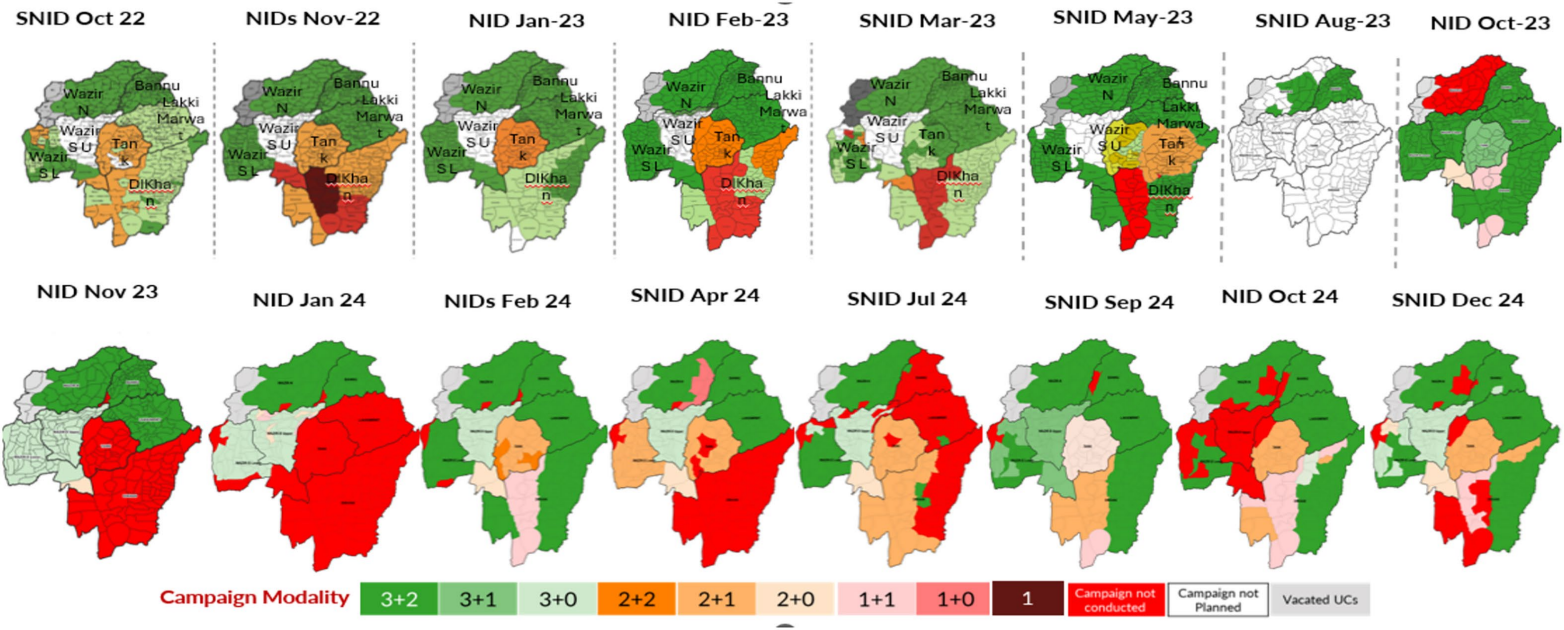
SIA implementation utilizing various security-led modalities, South Khyber Pakhtunkhwa, October 2022–December 2024. NID, National Immunization Days which are nationwide polio immunization campaigns; SNID, Sub-national Immunization Days which are polio immunization campaigns targeting high risk sub-national geographies. The numbers noted as campaign modality (3 + 2, 3 + 1, etc.) reflect the number of days polio campaigns were conducted with the first number before the plus sign reflecting actual campaign days and the second number after the plus sign reflects catch-up days.

To maintain vaccinators’ safety without cancelling all SIAs, the polio program instituted a “staggered” implementation strategy. SIAs in South KP were conducted after the scheduled time assigned for campaigns in the rest of the province and the country. The campaign duration was also shortened sometimes, from the standard 5 days (3 days of house-to-house vaccination and 2 days of catch-up for children missed during the previous days) to 1 to 3 days (Figure 2). The specific schedules and duration of campaigns were decided at the lowest administrative levels (UC and area) depending on the security situation.

In South Waziristan Upper district where community boycotted polio campaigns, a fixed-site modality was adopted in November 2023 to enhance community acceptance. A total of 128 sites were identified for delivery of OPV, and community workers encouraged caregivers to bring children under 5 years of age independent of previous vaccination through routine immunization or campaigns.

To support frontline workers facing security challenges, the Pakistan polio program increased their remuneration in March 2022 and changed their status from Mobile Teams to Special Mobile Teams provided with additional logistical support for pre-, intra-, and post-campaign activities. However, during the reporting period, monitors reported fake finger marking incidents, where the teams in collusion with community members would falsely finger mark children who were not vaccinated. This led the program to adopt the Directly Observed Vaccination (DOV) strategy, in which an independent observer was deployed with each team to monitor vaccination and finger marking. This strategy was implemented in all districts of South KP, except for South Waziristan Upper. Finally, in June 2022, vaccine delivery changed from house-to-house to site-to-site to improve frontline workers’ safety. On average one site was selected for every five households for easy access for the community. In addition, parents who brought their children to the vaccination sites received incentives, such as soap and detergents.

### Special interventions to vaccinate children in hard-to-reach populations

The KP provincial government and the polio eradication program also implemented several strategies to administer polio vaccines during the study period to populations with difficult access because of security or social isolation.

#### Biker vaccination teams

Starting in September 2022, 29 vaccination teams provided with motorbikes followed and vaccinated children of high-risk mobile populations (HRMP), who travel for seasonal work, religious and security reasons within South KP, to other areas of KP province and to other provinces. These biker teams aimed to provide OPV to all children under 5 years and RI antigens to children below 2 years.

From September 2022 to December 2024, these Biker Vaccination Teams vaccinated 5,676 children with BCG, 186,752 with OPV, 18,858 with the first dose of pentavalent vaccine (Penta-1), 15,381 with Penta-2, 14,252 with Penta-3, 28,798 with Measles and 41,317 with IPV. District wise, the highest number of children vaccinated with OPV (*n* = 75,427), IPV (*n* = 13,550), Penta-1 (*n* = 5,899), and Penta-3 (*n* = 9,452) was reported in D. I. Khan. South Waziristan Upper reported the lowest number children vaccinated with OPV (*n* = 5,129) and IPV (*n* = 1,699). Lakki Marwat reported the highest number of children vaccinated against Measles (*n* = 9,192), while Tank reported the lowest (*n* = 879). The large variation in number of children vaccinated by district is due to differences in resource allocation, security issues, and high turnover of teams.

#### Permanent transit posts (PTP)

An additional strategy to vaccinate HRMP included the installation of transit vaccination posts in strategic locations at district borders, where OPV was administered to children below 10 years of age traveling between districts. Due to seasonal population movements, the number of PTPs fluctuated between 132 and 299 during the study period. The number of PTP also varied by district. As of December 2024, there were 23 in Bannu, 26 in Lakki Marwat, 11 in North Waziristan, 33 in DI Khan, 19 in Tank, 16 in South Waziristan Lower, and 11 in South Waziristan Upper. The number of children vaccinated at these PTPs reached 1,117,940 in 2022; 3,102,875 in 2023 and 3,088,704 in 2024.

#### Ring fencing vaccination

This strategy was first initiated in South Waziristan Upper in December 2022 following the cancellation of polio SIAs due to community boycott. A total of 31 ring vaccination posts were placed at key points in the outskirts of the district to provide OPV to individuals entering and exiting the district. Initially individuals of all ages were vaccinated, but since March 2023 onwards vaccination was restricted to children under 10 years of age. The teams vaccinated 386,800 children <10 years of age in 2022, 889,010 in 2023 and 1,010,508 in 2024 in these “ring vaccination posts”.

### PEI-EPI synergy initiatives

The polio program collaborated with the Federal Directorate of Immunization on initiatives aimed at addressing gaps in both polio and RI activities to improve population immunity to all vaccine-preventable diseases.

#### Fractional-dose inactivated polio vaccine (fIPV) campaigns

**Figure 3:**
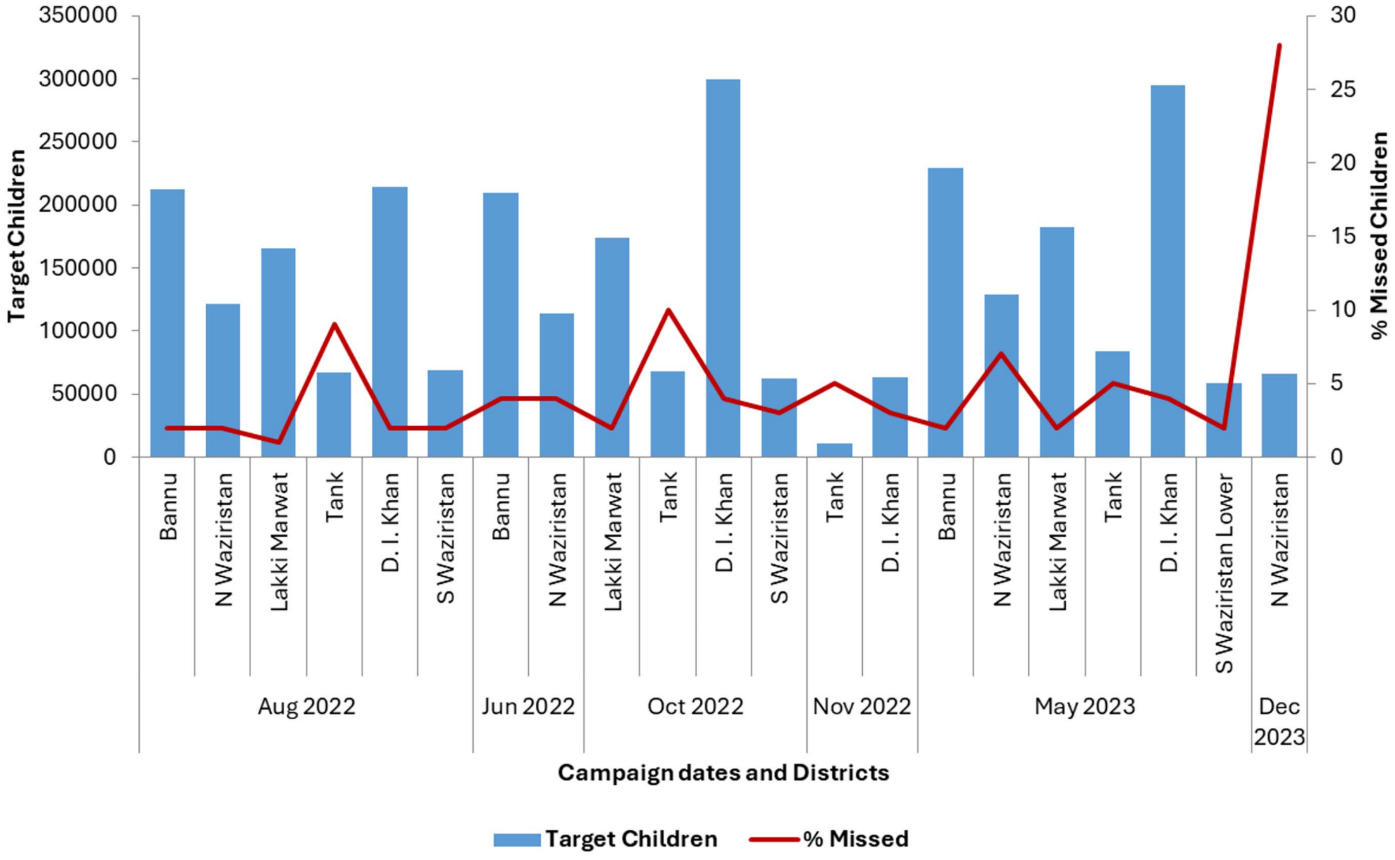
Number of children targeted, and percent targeted children missed during campaigns delivering fractional-dose inactivated polio vaccine (fIPV) conducted from August 2022 to December 2023 in South KP.

In response to increased detection of WPV1 during the first half of 2022 and due to the low IPV coverage in routine immunization coupled with low acceptance of OPV in some communities in the region, six fIPV focused campaigns were conducted in South KP from August 2022 to December 2023. Children aged 4–59 months were targeted for fIPV during these campaigns while complementary dose of OPV was given to children aged 0–59 months. To increase community acceptance of vaccines, detergents and soap were also provided. Less than 10% of target children missed fIPV or OPV during these campaigns in all districts except for North Waziristan, where the Utmanzai tribal community boycotted the December 2023 round (Figure 3).

#### Zero dose coverage

A total of 315,223 zero-dose children aged 0–23 months were identified during the 19 SIAs conducted in South KP during the study period. Of these, 64% (*n* = 200,278) were followed up by the EPI team and started on a RI schedule using a variety of strategies such as Reaching the Unreached (RUR), Extended Outreach Activities (EOAs), Big Catch-up (BCU), and Health Camps. The proportion of zero-dose children identified by polio frontline workers decreased over time from 2022 to 2023 in some districts. The proportion of those zero-dose children who received RI antigens also varied by district. Tank and D. I. Khan reported >70% of zero-dose children receiving RI antigens, whereas other districts reached less than 50% of children. South Waziristan was split into two districts: South Waziristan Upper and South Waziristan Lower in April 2022. In South Waziristan Upper the Utmanzai tribal community boycotted polio campaigns from July 2022 to November 2023 leading to a low number of zero dose children identified during polio SIAs in that district (Table 2).

**Table 2:** Number of zero dose children identified during polio immunization campaigns and proportion of those children who received routine immunization (RI) antigens through special strategies, South KP, 2022–2024.

Districts	2022		2023		2024	
	Number of zero dose children identified	Percent receiving RI antigens	Number of zero dose children identified	Percent receiving RI antigens	Number of zero dose recorded children	Percent receiving RI antigens
D. I. Khan	32,173	73%	20,297	72%	26,548	64%
Tank	7,106	67%	5,334	89%	5,999	61%
South Waziristan Lower	8,516	50%	53,77	50%	3,609	44%
South Waziristan Upper	0	0%	20	100%	564	73%
Bannu	34,868	52%	29,612	73%	30,327	56%
Lakki Marwat	23,145	57%	23,837	60%	25,393	70%
North Waziristan	14,391	66%	8,638	62%	9,469	68%
Total	120,199	61%	93,115	68%	101,909	63%

#### Extended outreach activities (EOA) and big catch-up (BCU)

The EOAs were implemented by an EPI team with support of polio staff between polio SIAs, while the BCU was a time-limited activity conducted to address specific RI gaps. These initiatives delivered RI vaccines to children below 2 years of age who had been identified during SIAs as zero-dose, missing some doses or due for a vaccine dose. Eight EOAs and one Big catch-up activity were conducted, targeting 756,669 zero-dose, due, and defaulter children, with 78% of these children vaccinated. Penta-1/OPV-1 coverage during these activities reached 86%, while Penta-3/OPV-3 coverage was 80% (Table 3). EOAs and BCUs proved effective in reaching children who had been missed by RI services increasing vaccination rates in South KP.

**Table 3:** Target children and proportion vaccinated with several RI antigens during extended outreach activities (EOA) and the big catch-up (BCU), South KP, June 2022 to October2024.

Activity	Total number of target children	Coverages (%)				
		Overall coverage	Penta-1 / OPV1	Penta-3 / OPV3	IPV-1	IPV-2
EOA Jun-22	37,434	68%	64%	73%	92%	89%
EOA Sep-22	79,202	84%	110%	87%	90%	90%
EOA Nov-22	43,022	76%	92%	81%	82%	80%
EOA Jan-23	69,033	85%	99%	88%	92%	87%
EOA May-23	63,945	86%	97%	89%	90%	89%
EOA Oct-23	71,288	85%	100%	86%	86%	82%
EOA Feb-24	67,367	78%	89%	78%	80%	75%
EOA May-24	69,192	79%	81%	69%	71%	65%
BCU Oct-24	216,380	71%	70%	71%	71%	66%
Total	756,669	78%	86%	80%	81%	77%

The OPV/Penta 1 coverages were >100% due to denominator issues in these security-compromised pockets and Polio Plus Strategy (Soap, detergents, balloons, whistle and Panadol syrup provision) that attracted people from neighboring settlements that were not included in the initial target.

#### Reaching the unreached (RUR)

Three rounds of special vaccination campaigns delivering multiple antigen RI antigens to children under 5 years old were implemented in 69 high-risk union councils during July 2023 to April 2024. Details of this initiative are reported in a separate manuscript (15).

#### Health camps

From January 2022 to December 2024, the KP provincial health department implemented 10,366 health camps in the South and North Waziristan districts to address suboptimal healthcare services delivery and community resistance. The integration of services allowed vaccination of 6,284 zero-dose children and 2,487 children from families who had refused RI services. A total of 49,117 and 19,628 children under 2 years received Penta-1/OPV-1 and Penta-3/OPV-3, respectively, while 54,336 and 232,961 children under 5 received IPV and OPV, respectively.

## Discussion

The Pakistan polio program devised several strategies during 2022–2024 to tackle complex security challenges and community mistrust, aiming to expand vaccination activities, close immunity gaps, and reduce the burden of paralytic polio, in the two divisions of South KP region. These strategies included staggered polio SIAs, campaigns delivering fIPV, special campaigns to reach underserved children, enhanced outreach RI activities, and health camps. The use of biker teams, ring fencing, and permanent transit post also helped in overcoming inaccessibility, high population mobility, and difficult terrains that hinder traditional vaccination delivery mechanisms in the region. These efforts in South KP demonstrate the critical role of innovative, context-specific strategies in addressing unique challenges limiting immunization services in high-risk and security-fragile areas. Several lessons were learned with the implementation of each of these strategies.

The security-guided approach to implementing SIAs enabled the polio program to protect frontline workers while continuing to vaccinate children in South KP. Staggering instead of cancelling vaccination campaigns in areas with security concerns allowed vaccination in large areas of a district not affected by insecurity. However, limitations in monitoring and post campaign assessments in those areas with insecurity made it difficult to confirm the quality of SIAs. That said, the LQAS assessment found that only four out of the 19 campaigns in South KP did achieve the national benchmark of ≥80% lots passing at ≥90% coverage. LQAS could not be done in most areas of South Waziristan Upper district where only two UCs were assessed after SIA resumption following 16 month hiatus and it was difficult to carry out re-vaccination in areas that had failed LQAS surveys following short (1- or 2 day) SIAs, which led to accumulation of missed children over multiple campaigns (16). Furthermore, the lack of synchronization of campaigns in South KP with the rest of the country or even within the region, made it very difficult to identify and vaccinate children who were traveling during campaigns. These children that fall between the cracks by virtue of their mobility increase the burden of susceptible population in the region. On the other hand, because of these children on the move, coverage in one area may be inflated while other areas may not reach the expected targets. Because of these negative repercussions on campaign quality, the program can consider limiting staggered campaigns to areas where this approach is essential to ensure vaccinators’ safety. On the other hand, it would be important to incorporate standardized data from novel strategies in the local polio data systems, including SIA operational modality adopted by each union council and LQAS assessments that could not be conducted due to insecurity. Restricted LQAS implementation and incomplete monitoring in high-risk districts limited independent validation of campaign quality, potentially affecting the accuracy of reported coverage estimates. Challenges in maintaining coverage quality and monitoring in conflict-affected settings have been documented in both global and Pakistan-specific literature (11, 13).

The biker teams were very effective at reaching communities that are not accessible through standard modes of operation and/or transportation, and that were often missed by routine immunization services and campaigns. Their work was particularly crucial in South Waziristan Upper after all RI activities and SIAs were suspended for over a year (July 2022 to November 2023) because of community boycotts. The use of motorbikes allowed vaccinators to cover vast hard-to-reach areas, ensuring children receive timely vaccinations. The variability in the number of children reached across districts of South KP highlighted how logistical barriers or insecurity affected some of the teams’ ability to consistently reach their target populations.

Ring fencing and PTP strategies in South KP focused on reaching security-compromised and mobile populations and were inspired by the examples from other countries (17). Vaccination posts established at checkpoints along major travel routes and around areas inaccessible to traditional vaccination teams, ensured vaccine availability for populations that are constantly moving, such as seasonal workers or internally displaced persons. In 2014, Nigeria transit vaccination teams proved decisive for reaching children missed in previous campaigns and in India, mobile vaccination teams reached highly mobile populations at bus depots, train stations, and other transit points (18, 19).

Due to the low IPV coverage in routine immunization, low acceptance of OPV in some communities, and reduction in number of campaigns for security reasons, the polio program initiated periodic targeted IPV campaigns (20). To reduce vaccination costs, and considering that the campaign would deliver a booster dose to most of the target children, fractional-dose IPV (fIPV) containing one fifth of the usual dose, was delivered together with OPV and small tokens (soap, detergent) in six campaigns during the study period (21). These campaigns showed that use of fIPV in campaigns is feasible, and can reach acceptable coverage, based upon the small proportions of children missed observed in most districts in South KP. Targeted fIPV campaigns have also been successfully conducted in countries like Somalia, Nigeria, and India, to help close immunity gaps to all three poliovirus serotypes in high-risk hard-to-reach populations without causing constraints in global IPV supply (22–24).

The Pakistan Polio Eradication Initiative (PEI) and the Expanding Program for Immunization (EPI) started the PEI-EPI Synergy initiative in 2015 to address gaps in both programs. It was piloted in 16 districts and later expanded to all high-risk districts in the country. In South KP, vaccination with polio and other antigens using “enhanced outreach,” “reaching the unreached” and “the big catchup” strategies, resulted in a notable reduction in zero-dose, under-immunized and missed children in the region (25, 26). These strategies were reinforced by activities tailored to specific gaps such as integrated health camps, targeted outreach sessions in underserved areas, community-centered communication activities and enhanced monitoring and supervision of RI services (27, 28). These synergy initiatives contributed to overall improvement in vaccination coverages and 64% of identified zero-dose children were linked to the RI program. Improving coverage of OPV and IPV in RI contributed to increasing polio immunity and reducing transmission. RI efforts and surveillance systems benefited from logistical support provided by the PEI, leading to higher coverage rates for critical vaccines, such as Pentavalent, Measles and Rubella, and IPV (29).

### Lessons learned

Key lessons from these interventions include the importance of community-centered interventions and culturally appropriate engagement to improve vaccine acceptance, particularly among underserved communities. In this context, several operational components were particularly effective in reducing persistently missed children and improving uptake of both campaign and routine immunization services. Biker teams and extended outreach approaches were useful for reaching scattered, remote, and repeatedly missed settlements while transit point vaccinations reached mobile and transient populations moving along key corridors. Ring fencing vaccination enabled rapid containment and provided opportunities to overcome security-related disruptions.

Microplanning grounded in real-time data, mobile outreach, effective targeting of high-risk locations and improved operational efficiency are also critical in ensuring success of interventions in areas with limited access to health services because of conflict or geographical isolation (30, 31). The integration of polio-specific platforms with RI not only increased coverage but also supported the sustainability of immunization efforts. These experiences highlight the critical importance of integrated, flexible, and context-specific approaches to immunization in fragile and conflict-prone regions. Interventions like those implemented in South KP have also previously shown good results in improving childhood survival and reducing the burden of vaccine preventable diseases in places like Angola and Nigeria (30).

### Limitations

Our study is subject to some limitations. First, lack of proper denominators for some of the interventions, such as biker teams, transit teams, ring strategy, and health camps, made it difficult to measure the achievements against any specific target. This may lead to inflated coverage and sense of false optimism towards achieving polio eradication and may negatively affect decision making. Second, the number of children reached through the described strategies were influenced by seasonal population movement, a fluid security situation and resources constraints. Finally, the same logistical challenges interfering with vaccination activities prevented effective monitoring and evaluation activities that could provide a rigorous assessment of the impact of these interventions.

## Conclusion

Polio eradication in Pakistan requires context-specific, locally tailored vaccination strategies rather than a “one size fits all” approach. Success depends on adopting innovative interventions, engaging community and administrative leaders, and coordinating with other immunization programs to ensure acceptance and coverage. Despite the best efforts by the polio program, insecurity, operational gaps, population movement, and community demand for other services continue to hinder vaccination efforts in South KP. Continued investment, sustained political will, cross border collaboration, ensuring safety and motivation of frontline workers, and ongoing evaluation of program performance, will be crucial for maintaining momentum in this critical public health initiative. Strengthening synergy between polio and routine immunization not only advances polio eradication but also contributes to broader health improvements in Pakistan.

## Data Availability

The data analyzed in this study is subject to the following licenses/restrictions: governmental restrictions. Requests to access these datasets should be directed to eocpakhtunkhwa@gmail.com.
